# NEFA Promotes Autophagosome Formation through Modulating PERK Signaling Pathway in Bovine Hepatocytes

**DOI:** 10.3390/ani11123400

**Published:** 2021-11-28

**Authors:** Yan Huang, Chenxu Zhao, Yaoquan Liu, Yezi Kong, Panpan Tan, Siqi Liu, Fangyuan Zeng, Yang Yuan, Xinwei Li, Guowen Liu, Baoyu Zhao, Jianguo Wang

**Affiliations:** 1 College of Veterinary Medicine, Northwest A&F University, Yangling 712100, China; hy0016@nwafu.edu.cn (Y.H.); cxzhao@nwafu.edu.cn (C.Z.); liuyq0703@nwafu.edu.cn (Y.L.); kongyezi2019@nwafu.edu.cn (Y.K.); Tanpp@nwsuaf.edu.cn (P.T.); liusiqi@nwafu.edu.cn (S.L.); zengfy@nwafu.edu.cn (F.Z.); 18821618677@nwafu.edu.cn (Y.Y.); zhaobaoyu12005@nwafu.edu.cn (B.Z.); 2 College of Veterinary Medicine, Jilin University, Changchun 130062, China; lixinwei100@jlu.edu.cn (X.L.); gwliu@jlu.edu.cn (G.L.)

**Keywords:** non-esterified fatty acids, protein kinase R-like endoplasmic reticulum kinase, autophagy, dairy cows

## Abstract

**Simple Summary:**

Negative energy balance can lead to the mobilization of large amounts of body fat. A large amount of non-esterified fatty acids (NEFA) produced by lipolysis not only can be used for energy generation by β-oxidation in the liver but can also act as a potential regulator of lipid metabolism. The present study provides evidence that NEFA can activate hepatocyte autophagy through the protein kinase R-like endoplasmic reticulum kinase (PERK) signaling pathway. Autophagy has been reported to contribute to lipid metabolism through promoting the breakdown of intracellular lipids. These findings enable a better understanding of the redistribution and utilization of energy during the perinatal period of dairy cows.

**Abstract:**

During the perinatal period, the abnormally high plasma non-esterified fatty acids (NEFA) concentration caused by the negative energy balance (NEB) can impose a significant metabolic stress on the liver of dairy cows. Endoplasmic reticulum (ER) stress is an important adaptive response that can serve to maintain cell homeostasis in the event of stress. The protein kinase R-like endoplasmic reticulum kinase (PERK) pathway is the most rapidly activated cascade when ER stress occurs in cells and has an important impact on the regulation of hepatic lipid metabolism and autophagy modulation. However, it is unknown whether NEFA can affect autophagy through modulating the PERK pathway, under NEB conditions. In this study, we provide evidence that NEFA treatment markedly increased lipid accumulation, the phosphorylation level of PERK and eukaryotic initiation factor 2α (eIF2α), and the expression of glucose-regulated protein 78 (Grp78), activating transcription factor 4 (ATF4), and C/EBP homologous protein (CHOP). More importantly, NEFA treatment can cause a substantial increase in the protein levels of autophagy-related gene 7 (ATG7), Beclin-1 (BECN1), sequestosome-1 (p62), and microtubule-associated protein 1 light chain 3 (LC3)-II, and in the number of autophagosomes in primary bovine hepatocytes. The addition of GSK2656157 (PERK phosphorylation inhibitor) can significantly inhibit the effect of NEFA on autophagy and can further increase lipid accumulation. Overall, our results indicate that NEFA could promote autophagy via the PERK pathway in bovine hepatocytes. These findings provide novel evidence about the potential role of the PERK signaling pathway in maintaining bovine hepatocyte homeostasis.

## 1. Introduction

During the perinatal period, dairy cows have been found to display a negative energy balance (NEB) [1]. The high level of non-esterified fatty acids (NEFA) in the plasma induced by NEB can serve as an important trigger of fatty liver and ketosis in dairy cows [2]. NEFA are an important energy source for perinatal dairy cows and are also necessary for the synthesis of milk fat [3]. However, when the lipid synthesis induced by NEFA exceeds the rate of fatty acid oxidation and secretion in the liver, triglycerides (TG) can be stored as lipid droplets within the hepatocytes, which, in turn, can lead to a fatty liver condition [4].

It has been shown that NEFA-induced lipotoxicity can elicit several stress-related responses, such as endoplasmic reticulum (ER) stress and oxidative stress [5,6,7]. The perturbations in ER homeostasis can lead to increased accumulation of unfolded or misfolded proteins in the ER during cellular stress, which is collectively termed ER stress. The unfolded protein response (UPR) can be thereafter activated to alleviate the ER stress, thus promoting cell survival [8]. As one of the three branches that can constitute the UPR, the protein kinase RNA-like ER kinase (PERK) pathway is the most rapidly activated cascade during this process [9]. This pathway can not only significantly inhibit protein translation through affecting the phosphorylated eukaryotic initiation factor 2α (eIF2α) but can also stimulate the ER-associated degradation (ERAD) pathway by upregulating activating transcription factor 4 (ATF4) expression [10,11]. Therefore, the activation of the PERK signaling pathway could be of great significance for maintaining the homeostasis of the ER. In addition, as a transcription factor, ATF4 could actively participate in the activation of the autophagy signaling pathway to maintain hepatocyte homeostasis [12].

Autophagy is a catabolic process in which the damaged cytoplasmic organelles or cytosolic components are degraded by lysosomal proteases, and it can contribute to modulating lipid metabolism by directly regulating lipid degradation [13,14]. A number of previous studies have shown that dairy cows with mild fatty liver show a more active lipid metabolism and enhanced autophagy, which serve as adaptive mechanisms of dairy cows during the perinatal period [15,16]. Therefore, when the liver is exposed to a high blood concentration of NEFA, autophagy plays an important role in maintaining the stability of hepatocytes, thereby providing nutrients and alleviating lipid accumulation. Our previous study showed that NEFA can activate the PERK-eIF2α signaling pathway to regulate lipid metabolism in hepatocytes [6]. However, it is unclear whether NEFA could mediate the activation of the PERK-eIF2α signaling pathway to affect hepatic autophagy in dairy cows. The purpose of this research was thus to study the potential effect of the NEFA-mediated PERK-eIF2α signaling pathway on the process of autophagy by means of the addition of GSK2656157 (a pharmacological PERK phosphorylation inhibitor). The findings of this study provide novel insights into the prevention and treatment of fatty liver and ketosis during the perinatal period.

## 2. Materials and Methods

### 2.1. Isolation, Culture, and Treatment of Calf Primary Hepatocytes

Bovine primary hepatocytes were isolated from five Holstein calves by digesting the liver using perfusion of type IV collagenase as previously described [17]. The isolated hepatocytes were seeded onto a 6-well tissue culture plate at 1 × 10^6^ cells/mL in adherent medium (RPMI-1640 basic medium supplemented with 10% FBS, 10^−6^ M of insulin, 10^−6^ M of dexamethasone, and 10 μg/mL of vitamin C). After incubation for 4 h in 5% CO_2_ at 37 °C, the adherent medium was replaced with the growth medium (RPMI-1640 basic medium supplemented with 10% FBS), with the medium being changed every 24 h.

After 60 h of culture, the hepatocytes were starved in 3.6% Bovine Serum Albumin (BSA, Sigma, St Louis, MS, USA) for 6 h. The hepatocytes were treated with 1 μM of GSK2656157 (a specific inhibitor of PERK, HY-13820, MCE, Malta, NY, USA) for 1 h before addition of NEFA. The hepatocytes were then cultured for 5 h at 37 °C and 5% CO_2_. The NEFA stock (52.7 mM) solution was prepared as previously described [6].

### 2.2. Nile Red Staining

In order to determine the lipid accumulation in hepatocytes, the hepatocytes were first stained using Nile red (A606340, Sangon Biotech Co., Ltd., Shanghai, China). The hepatocytes were fixed in 4% formaldehyde solution for 30 min and stained with 1 mg/mL Nile red solution for 20 min at 37 °C, followed by nuclear staining with 4,6-diamidino-2-phenylindole (DAPI) (C1002, Beyotime Institute of Biotechnology, Beijing, China). The stained cells were observed under laser confocal microscopy (Carl Zeiss GmbH, Jena, Germany), and images were captured.

### 2.3. Protein Extraction and Western Blotting

The expression of the various autophagy-related proteins was analyzed by Western blotting. The total protein was extracted from the cultured hepatocytes using a total protein extraction kit (Beyotime Institute of Biotechnology, Beijing, China). Equal amounts of proteins were separated on 10% or 15% SDS-polyacrylamide gels and electrophoretically transferred onto polyvinylidine fluoride membranes. The blots were incubated with corresponding primary antibodies overnight at 4 °C, followed by incubation with horseradish peroxidase-conjugated anti-mouse or anti-rabbit secondary antibodies for 2 h (Sangon Biotech Co., Ltd.). Thereafter, immunoreactive bands were visualized using enhanced chemiluminescence solution (P0018FS, Beyotime Institute of Biotechnology, Beijing, China) and imaged using a simple protein imager (ProteinSimple, Santa Clara, CA, USA). Each sample was run in triplicate, and the experiment was repeated thrice. The protein gray intensity was quantified by Image-Pro Plus 6.0 (Media Cybernetics, Bethesda, MD, USA) and normalized to β-actin.

The following primary antibodies were used for Western blot analysis: phospho-PERK antibody (1:1000; catalog No. 3179, Cell Signaling Technology, MA, USA), glucose-regulated protein 78 antibody (Grp78, 1:1000; catalog BA2042, Boster Biological Technology, Wuhan, China), PERK antibody (1:1000; catalog No. 5683, Cell Signaling Technology, MA, USA), phospho-eIF2α antibody (1:1000; catalog Ab32157, Abcam, Cambridge, UK), eIF2α antibody (1:1000; catalog Ab115822, Abcam, Cambridge, UK), ATF4 antibody (1:1000; catalog OM108094, OmnimAbs, Shanghai, China), C/EBP homologous protein antibody (CHOP, 1:1000; catalog AC532, Beyotime Institute of Biotechnology, Beijing, China), autophagy-related gene 7 (ATG7, 1:1000; catalogue number AA820, Beyotime Institute of Biotechnology, Beijing, China), sequestosome-1(SQSTM1/p62, 1:1000; catalogue number AF0279, Beyotime Institute of Biotechnology, Beijing, China), microtubule-associated protein 1 light chain 3 (MAP1LC3/LC3, 1:1000; catalogue number AL221, Beyotime Institute of Biotechnology, Beijing, China), Beclin-1 (BECN1, 1:1000; catalogue number D40C5, Cell Signaling Technology, MA, USA), β-actin antibody (1:5000; AB0035; Abways, Shanghai, China).

### 2.4. MCherry GFP LC3B Transfection

To detect the autophagic flux, hepatocytes were transfected with mCherry-GFP-LC3B adenovirus (C3011, Beyotime Institute of Biotechnology, Beijing, China). The hepatocytes were grown on 24-well plates until they reached approximately 30–50% confluence at the time of infection. Briefly, the hepatocytes were transfected with the mCherry-GFP-LC3B adenovirus at an MOI of 10 for 36 h at 37 °C before treatment with NEFA. After treatment with NEFA for 5 h, the hepatocytes were fixed with 4% paraformaldehyde and analyzed using laser confocal microscopy (Carl Zeiss GmbH, Jena, Germany).

### 2.5. Transmission Electron Microscopy

The ultrastructural characteristics of hepatocytes were visualized by transmission electron microscopy. Briefly, bovine primary hepatocytes were pelleted by centrifugation at 2000 rpm for 2 min and fixed in 2.5% glutaraldehyde for 4 h at 12 °C. Thereafter, the samples were fixed in 1% osmium tetroxide for 1 h, dehydrated through an ethanol series, and embedded in epoxy resin. Subsequently, ultra-thin sections (60–80 nm) were double stained with uranyl acetate and lead citrate. The representative areas were cut and examined under a transmission electron microscope (HT7800, Hitachi, Tokyo, Japan).

### 2.6. Statistical Analysis

Each experiment was repeated independently at least 3 times. The data were analyzed using repeated measurement ANOVA followed by Sidak’s multiple comparisons test. A value of *p* < 0.05 was considered statistically significant, and *p* < 0.01 was considered highly significant. The results were expressed as the mean ± standard error of the mean (SEM).

## 3. Results

**Figure 1:**
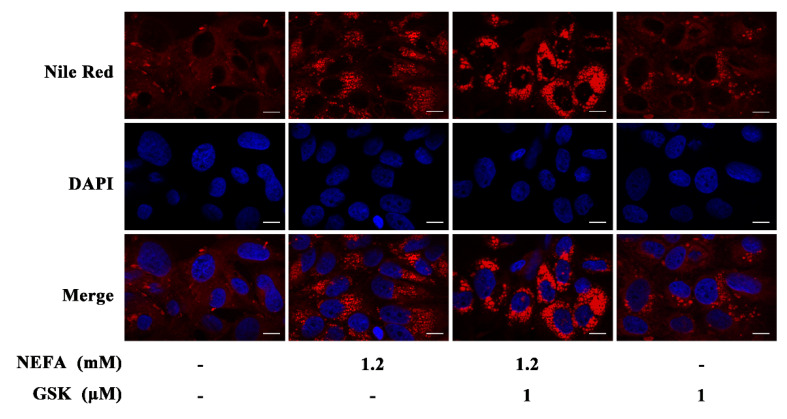
Fluorescence Nile red staining of primary bovine hepatocytes treated with the indicated concentrations of non-esterified fatty acids (NEFA) or GSK2656157. Scale bar: 10 μm.

As shown in Figure 1, NEFA treatment can remarkably increase the number of lipid droplets in the hepatocytes. Pretreatment with NEFA and GSK2656157 further increases the number of lipid droplets. This indicates that pretreatment with GSK2656157 exacerbated the NEFA-induced lipid accumulation.

**Figure 2:**
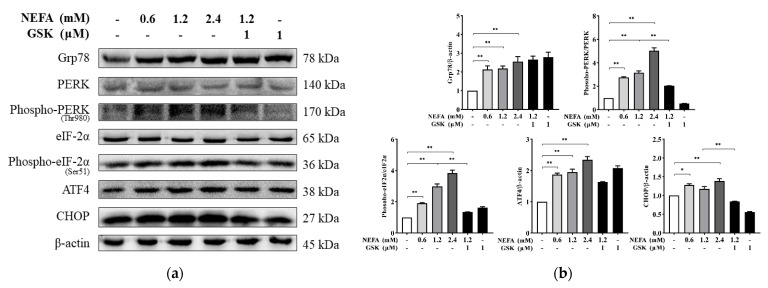
Status of the protein kinase RNA-like ER kinase (PERK) signaling pathway in primary hepatocytes. (**a**) Expression of hepatocyte PERK signaling pathway-related proteins was analyzed by Western blotting. (**b**) Quantitative analysis of hepatocyte proteins glucose-regulated protein 78 (Grp78), activating transcription factor 4 (ATF4), and C/EBP homologous protein antibody (CHOP), as well as phospho-PERK/PERK and phospho-eIF2α/eIF2α. Data are expressed as mean ± SEM (n = 3), * *p* < 0.05, ** *p* < 0.01.

To investigate the possible effect of NEFA on the activation of the PERK signaling pathway, Western blotting was conducted to detect the expression of the various proteins involved in the PERK signaling pathway. The protein expression of Grp78, ATF4, and CHOP and the phosphorylation levels of PERK and eIF2α were found to be substantially greater in hepatocytes treated with NEFA (*p* < 0.05 or *p* < 0.01, Figure 2). Pretreatment with GSK2656157 alleviated the NEFA-induced increase in protein expression of CHOP and the phosphorylation levels of PERK and eIF2α compared to 1.2 mM NEFA alone (*p* < 0.01).

**Figure 3:**
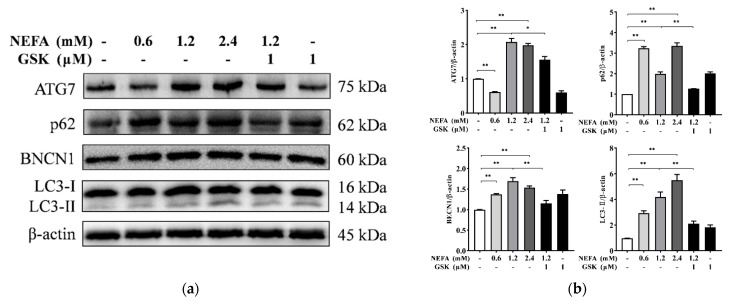
Status of autophagy in primary hepatocytes. (**a**) Expression of hepatocyte autophagy-related proteins was analyzed by Western blotting. (**b**) Quantitative analysis of hepatocyte proteins autophagy-related gene 7 (ATG7), sequestosome-1 (p62), beclin-1 (BECN1), and microtubule-associated protein 1 light chain 3 (LC3) II. Data are expressed as mean ± SEM (n = 3), * *p* < 0.05, ** *p* < 0.01.

The Western blot results show that the protein expression of ATG7, p62, BECN1, and LC3-II was increased in response to NEFA (*p* < 0.01, Figure 3). The protein abundance of ATG7 was lower in the hepatocytes treated with 0.6 mM NEFA compared to the control group (*p* < 0.01). However, compared to 1.2 mM NEFA alone, pretreatment with GSK2656157 significantly decreased the protein abundance of ATG7, p62, and LC3-II (*p* < 0.05 or *p* < 0.01).

**Figure 4:**
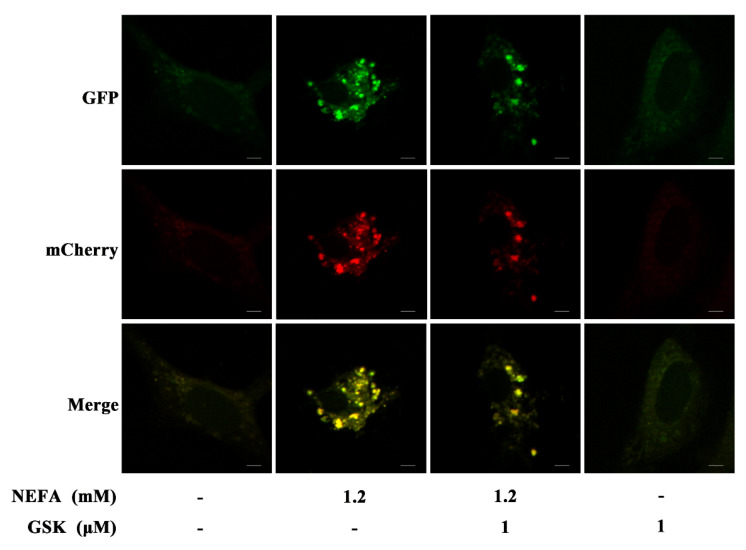
Representative images of hepatocytes transfected with mCherry-GFP-LC3B adenovirus in the presence of NEFA or GSK2656157 treatment. Scale bar: 4 μm.

To elucidate the possible effects of the NEFA-induced PERK-eIF2α signaling pathway on the autophagic process, the primary hepatocytes were transfected with mCherry-GFP-LC3B adenovirus. The formation of yellow dots (merged by mCherry and GFP fluorescence) indicated the autophagosomes, while the red dots (mCherry fluorescence) indicated the autophagolysosomes. As shown in Figure 4, NEFA treatment significantly increased the number of yellow dots representing the formation of autophagosomes. However, pretreatment with NEFA and GSK2656157 resulted in a substantial decrease in the number of yellow dots, thus suggesting that the process of autophagy was attenuated.

**Figure 5:**
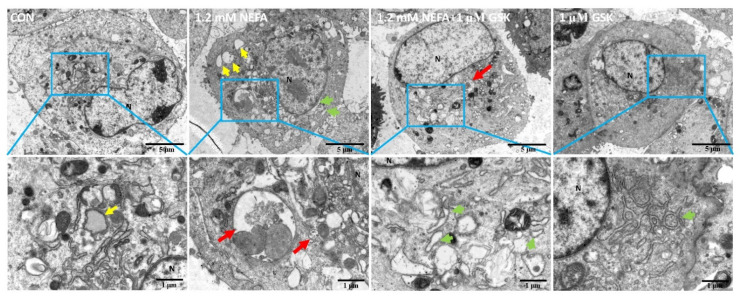
Morphological analysis conducted using transmission electron microscopy of bovine primary hepatocytes treated with NEFA or GSK2656157. CON, control; N, nucleus; red arrows represent autophagosomes; yellow arrowheads represent lipid droplets; green arrowheads represent endoplasmic reticulum (ER) expansion.

The effect of NEFA or GSK2656157 on autophagy in bovine primary hepatocytes was further investigated by transmission electron microscopy (Figure 5). We found that compared to the control group, there were relatively large numbers of autophagosomes (red arrows) in the hepatocytes treated with 1.2 mM NEFA, and the ER was dilated in shape (green arrowheads). Compared to 1.2 mM NEFA alone, there were fewer autophagosomes in the group pretreated with GSK2656157. Additionally, a more severely dilated ER was observed in the hepatocytes treated with GSK2656157.

## 4. Discussion

ER stress is considered a potent inducer of autophagy, and autophagy activation plays an important role in the cell survival process after ER stress [18]. A number of previous studies have shown that ER stress does not change the formation of autophagosomes, but it can potentially affect autophagosome–lysosome fusion [19,20]. Here, we show that the NEFA-induced PERK signaling pathway promotes an increase in the number of autophagosomes formed.

During the periparturient period, energy deficiency can promote the mobilization of fat, which can significantly increase the plasma NEFA concentration in dairy cows [21]. NEFA are not only potential substrates for liver lipid synthesis but can also regulate the expression of the various lipid metabolism genes [22]. Our results reveal that NEFA treatment increased the accumulation of lipids and activated the PERK signaling pathway in bovine hepatocytes. This observation can be explained by our hypothesis that when an excess of NEFA accumulates in the liver, NEFA-induced lipotoxicity can elicit several stress-related responses, such as those of ER and oxidative stress [5,7]. Mild ER stress has been found to be beneficial for cell survival, while intense ER stress can induce significant cell death. We used Nile red staining to demonstrate that activation of the PERK signaling pathway can significantly alleviate the NEFA-induced increase in TG. The content of lipids is primarily determined by a combination of lipid biosynthesis and decomposition. In our previous study, the NEFA-induced PERK signaling pathway was shown to be involved in enhanced lipid oxidation, which can effectively alleviate the lipotoxicity caused by NEFA [6]. Moreover, an increasing focus has been placed on the role of autophagy in liver steatosis because autophagy can effectively mediate lipid degradation [23,24]. Therefore, further research on the possible effects of the NEFA-mediated PERK-eIF2α signaling pathway on autophagy is required to better understand the underlying mechanisms of liver lipid metabolism in perinatal dairy cows.

Autophagy is an evolutionarily conserved mechanism that allows cells to degrade various cellular components and eliminate damaged organelles to maintain cellular homeostasis and energy balance [25]. ATG7 and BECN1 are key factors reported to mediate the initiation of autophagy and are involved in the conversion of LC3-I to LC3-II and the membrane extension of autophagosomes [26,27]. LC3-II and p62 can serve as vital markers for autophagy and are usually used to monitor the process of autophagy [28,29]. Our results indicate that NEFA can promote the protein expression of ATG7, p62, BECN1, and LC3-II in hepatocytes and increase the number of autophagosomes (Figure 3 and Figure 4), which clearly demonstrates that NEFA promotes autophagosome formation in hepatocytes. In line with our results, Chen et al. indicated that enhanced formation and degradation of autophagosomes were found in the liver of cows with mild fatty liver during the perinatal period [15]. Moreover, Li et al. previously suggested that activation of autophagy and an increase in the number of autophagosomes were found in the mammary tissues of cows with hyperketonemia [30]. Overall, autophagy can actively participate in the process of recycling and remobilizing nutrients in cells that serves as an adaptive mechanism for NEB.

Autophagy is a complex cellular process that involves the activation of several signaling pathways. A previous study in mice showed that the PERK-eIF2α signaling pathway can regulate the Atg5–Atg12 complex through ATF4, thereby regulating the occurrence of autophagy [12]. Moreover, PERK-eIF2α phosphorylation has also been reported to be involved in the conversion of LC3-I to LC3-II [31]. The PERK-eIF2α-ATF4 pathway has been found to be involved in the regulation of BECN1, which plays a vital role in the formation, elongation, and degradation of autophagosomes [12,32]. These studies have clearly established a positive role of the PERK-eIF2α pathway in regulating the transcription of autophagy-related genes. However, it remains unclear whether NEFA can also activate autophagy by the PERK signaling pathway when exposed to high serum NEFA concentrations during the perinatal period of dairy cows. In this current study, we demonstrated that the augmentation effect of NEFA-induced autophagy-related protein expression was significantly inhibited by GSK2656257 (a PERK inhibitor), which indicated that the NEFA-mediated PERK signaling pathway promoted autophagy in bovine hepatocytes. However, the specific mechanisms through which the PERK signaling pathway regulates autophagy in bovine hepatocytes remain unclear. A body of growing evidence indicates that peroxisome proliferator-activated receptor-α (PPARα) is a key regulator of lipid metabolism and could promote the expression of the β-oxidation-related gene carnitine palmitoyltransferase 1A; this could also induce autophagic lipid degradation, or lipophagy [33,34,35]. Our previous study demonstrated that the NEFA-induced PERK-eIF2α signaling pathway can effectively promote the transcriptional activity of PPARα [6]. The PERK-eIF2α signaling pathway might thus modulate the process of autophagy by mediating the transcriptional activation of PPARα.

A close relationship exists between lipid metabolism and autophagy. Autophagy can effectively reduce lipid toxicity in a number of animal models of disease [36]. However, in dairy cows with severe fatty liver, excessive fat infiltration in the liver of dairy cows can potentially impair autophagy activation, which may further aggravate lipid accumulation and thus lead to extensive cell damage and inflammation [37]. Moreover, in separate studies on humans and mice, it has been found that blocked macroautophagic flux can significantly induce ER stress and oxidative stress and further aggravate liver steatosis and injuries [38,39]. The increase in autophagosomes can thus potentially promote energy redistribution in hepatocytes, thereby favoring the breakdown of lipids, meaning that the liver of dairy cows can better adapt to the high serum NEFA concentration during the perinatal period. Thus, PERK-mediated autophagy might be a potential therapeutic target for the prevention and treatment of lipid accumulation induced by high NEFA concentrations in early-lactating dairy cows. Future studies in transition cows should examine how autophagy could be involved in or mediate lipid metabolism, providing insights which could help to significantly improve our understanding of hepatic lipid metabolism and minimize the incidence of fatty liver diseases.

## 5. Conclusions

In this study, we clearly demonstrated that the NEFA-mediated PERK signaling pathway can significantly stimulate autophagosome formation in bovine hepatocytes. These findings can contribute further to our understanding about the possible role of the PERK signaling pathway in maintaining homeostasis in bovine hepatocytes. Therefore, together with our previous findings, it can be concluded that during the perinatal period of dairy cows, the activation of the PERK signaling pathway can markedly enhance the hepatic adaptive response to high plasma NEFA concentrations, effectively regulating hepatic lipid metabolism to significantly reduce lipid accumulation.

## Data Availability

The raw data supporting the conclusions of this article will be made available by the authors, without undue reservation.
